# Segurança do Agente de Contraste SF_6_ (SonoVue^Ⓡ^) no Ecocardiograma sob Estresse Farmacológico

**DOI:** 10.36660/abc.20200475

**Published:** 2021-09-16

**Authors:** Rogerio Gomes Furtado, Daniela do Carmo Rassi, Luciano Henrique Melato, Ana Caroline Reinaldo de Oliveira, Paula Meneses Nunes, Priscila Elias Baccelli, Sara Camila de Oliveira Santos, Victor Emanuel Santos, Luiz Rassi, Colandy Godoy Nunes

**Affiliations:** 1 Centro de Diagnóstico por Imagem Goiânia GO Brasil Centro de Diagnóstico por Imagem (CDI), Goiânia, GO – Brasil; 2 Universidade Federal de Goiás Goiânia GO Brasil Universidade Federal de Goiás, Goiânia, GO – Brasil; 3 Hospital Santa Casa de Misericórdia de Goiânia Goiânia GO Brasil Hospital Santa Casa de Misericórdia de Goiânia, Goiânia, GO – Brasil

**Keywords:** Ecocardiograma sob Estresse, SonoVue^®^, Segurança

## Abstract

**Fundamento:**

Em 2007, a Food and Drug Administration (FDA) determinou revisões sobre segurança dos agentes de contraste ecocardiográfico (ACE) disponíveis no mercado após relatos de mortes. Ao longo desses anos, diversos estudos comprovaram a segurança dos ACE, porém com poucos estudos relacionados ao SonoVue^®^.

**Objetivos:**

Avaliar a segurança do SonoVue^®^ durante o ecocardiograma sob estresse farmacológico (EEF) por meio da análise da incidência de reações alérgicas e da comparação entre os grupos quanto ao surgimento de arritmia, efeitos colaterais menores e eventos adversos.

**Métodos:**

Estudo observacional, prospectivo, no qual 2.346 pacientes foram submetidos ao EEF e divididos em dois grupos: grupo 1 com ACE (n=1.099) e grupo 2 sem ACE (n=1.247). Os pacientes foram avaliados durante o EEF – 24 horas e 30 dias. Foi definido p significativo quando <0,05.

**Resultados:**

O grupo 1 apresentou efeitos colaterais mais leves, como cefaleia (5/0,5% *vs.* 19/1,5%, p=0,012) e hipertensão reativa (3/0,3% *vs* . 19/1,5%, p=0,002), menos arritmias como extrassístoles ventriculares (180/16,4% *vs* . 247/19,8%, p=0,032) e taquicardia paroxística supraventricular (2/0,2% *vs* . 15/1,2%, p=0,003), assim como nenhum evento adverso como infarto agudo do miocárdio (IAM) e óbito. No grupo 2, um paciente apresentou IAM <24h (1/01%) e dois óbitos <30 dias (2/0,1%). Urticária relacionada ao SonoVue^®^ foi observada em 3 (0,3%) pacientes sem reação anafilática.

**Conclusão:**

SonoVue^®^ demonstrou segurança durante o EEF, não sendo observados morte, IAM ou reação anafilática. Observou-se menor incidência de efeitos colaterais mais leves e arritmias no grupo que utilizou o ACE, assim como baixa incidência de reações alérgicas leves.

## Introdução

A ecocardiografia é reconhecida como um exame seguro, não invasivo e altamente reprodutível na análise das estruturas anatômicas e funcionais do coração. Entretanto, até 30% dos estudos são tecnicamente difíceis devido à baixa qualidade de imagem,^1 , 2^ principalmente em pacientes obesos, com deformidades torácicas e com doença pulmonar obstrutiva crônica.^3 , 4^

Em 1997, a Food and Drug Adminstration (FDA) aprovou o uso dos agentes de contraste ecocardiográfico (ACE), com o objetivo de melhorar a acurácia diagnóstica do método após revisar os dados quanto a sua segurança.^5^ Estudos de fase III demonstraram segurança e, consequentemente, os ACE foram aprovados e liberados para delineamento das bordas endocárdicas.^6 , 7^

Contudo, em outubro de 2007, a FDA interrompeu seu uso após 11 óbitos, que foram temporalmente relacionados ao emprego dos ACE.^8^ Após revisão em 2008, a FDA liberou novamente o uso dos ACE, porém contraindicados em pacientes com desvios ( *shunts* ) intracardíacos conhecidos ou com hipersensibilidade ao perflutreno.^9^

A segurança dos ACE foi documentada ao longo desses anos em diversos cenários clínicos, como em pacientes com hipertensão pulmonar, desvios *(shunts* ) intracardíacos e pacientes críticos. Grandes estudos levaram a mudanças na FDA quanto à liberação do uso dos ACE nesses cenários descritos, além de documentarem a importância do seu emprego na melhoria dos resultados dos pacientes. Ensaios clínicos também demonstraram segurança e eficácia dos ACE na ecocardiografia sob estresse físico e farmacológico, bem como seu emprego na avaliação da perfusão miocárdica.^10^

O ecocardiograma sob estresse farmacológico (EEF) é uma modalidade estabelecida para o diagnóstico da doença arterial coronariana (DAC), tendo segurança demonstrada em vários estudos.^11^ A utilização do ACE no EEF teve seu uso consolidado ao longo dos anos – inicialmente, para delineamento das bordas endocárdicas e, posteriormente, para o estudo de perfusão miocárdica.^10^ O uso dos ACE é indicado quando dois ou mais segmentos do ventrículo esquerdo (VE) não são visibilizados de forma adequada.^9 , 10^

Em 2013, a Agência Nacional de Vigilância Sanitária (Anvisa) liberou o uso do SF_6_/hexafluoreto de enxofre (SonoVue^®^) no Brasil. Apesar da sua segurança já demonstrada previamente, existem na literatura poucos estudos relacionando seu emprego e o surgimento de eventos adversos.^12^

## Métodos

### Descrição do estudo

Estudo observacional, prospectivo, descritivo, aprovado pelo Comitê de Ética e Pesquisa do Hospital de Urgências de Goiânia (HUGO/protocolo de número 31442100 pela Plataforma Brasil) em que foram avaliados através do EEF pacientes encaminhados para estratificação de risco de DAC. Os pacientes foram incluídos após a assinatura do TCLE.

No EEF dos pacientes em que não se visibilizava adequadamente dois ou mais segmentos do VE, foi acrescida a infusão de SonoVue^®^ para melhor delineamento das bordas endocárdicas.^9 , 10^

Os pacientes foram divididos em dois grupos, sendo o grupo 1 constituído de pacientes submetidos ao EEF com dobutamina-atropina com ACE SonoVue^®^ e o grupo 2, de pacientes submetidos ao EEF com dobutamina-atropina sem ACE.

Foram excluídos do estudo pacientes com história de reação alérgica ao ACE. Informações clínicas, antropométricas, fatores de risco (FR) para DAC, dados ecocardiográficos, presença ou ausência de arritmias, eventos adversos e reações alérgicas com menos de 30 minutos da realização do exame foram coletados.

Os pacientes do grupo 1 foram avaliados clinicamente quanto à presença de sinais e sintomas de reação alérgica nos primeiros 30 minutos após o exame de forma presencial. Após 24 horas, os pacientes foram avaliados de forma presencial ou através de ligações telefônicas.

Para avaliação de eventos adversos, como infarto agudo do miocárdio (IAM) e morte (24h e 30 dias), os pesquisadores fizeram ligações telefônicas para todos os pacientes, em ambos os grupos. Foram excluídos da pesquisa os pacientes que não atenderam às ligações telefônicas (3 ligações em dias distintos) ou que não retornaram ao consultório dos cardiologistas e ao Centro de Diagnóstico por Imagem (CDI).

### Avaliação ecocardiográfica

O EEF foi realizado em aparelhos de ecocardiografia EPIQ (Philips Ultrasound Systems^®^, Andover, MA, EUA). Os exames foram realizados por ecocardiografistas com o mesmo treinamento, de forma padronizada e uniforme, de acordo com as recomendações da *American Society of Echocardiography* (ASE).^13^

Os pacientes foram submetidos, inicialmente, ao ecocardiograma basal, com aquisição das medidas lineares das estruturas cardíacas e dos fluxos valvares. Para a avaliação da fração de ejeção do ventrículo esquerdo (FEVE), foram utilizados os métodos de Teichholz ou de Simpson, dependendo da extensão da alteração da contração segmentar. Em alguns casos, não se mediu o diâmetro sistólico final quando utilizado o método de Simpson para o cálculo da FEVE.^13 , 14^ Após aquisição das imagens no estágio basal (planos paraesternal longitudinal, transversal, apical quatro, três e duas câmaras), foi iniciada a infusão endovenosa de dobutamina, com dose inicial de 5μg/kg/min, com incrementos de doses a cada 3min para 10, 20, 30 e 40μg/kg/min. Foi administrada atropina em doses de 0,25mg, a cada 1min, até a dose máxima acumulativa de 2mg, caso o paciente não apresentasse sinais ecocardiográficos de isquemia miocárdica e não tivesse atingido a frequência cardíaca de, no mínimo, 100bpm no estágio de 20μg/kg/ min.

Para a aquisição de imagens específicas com o ACE, foram empregadas as técnicas de modulação da amplitude de pulso e a inversão do pulso do ultrassom (fundamental e harmônica), com baixo índice mecânico (<0,20) associado ou não com um breve pulso de ultrasom com alto índice mecânico ( *flash* ) para uniformizar a opacificação das bordas endocárdicas.^10^

Foi padronizado um tempo de 30min de monitoramento após o término da infusão para avaliar: os efeitos adversos, presença de sinais e sintomas de reação alérgica (grupo 1) e retorno da frequência cardíaca (FC) ao valor inferior a 100 batimentos por minuto.^11^

Durante o EEF, os pacientes foram mantidos sob monitoramento contínuo (medidas de pressão arterial, FC e eletrocardiograma em 12 derivações). A sintomatologia dos pacientes foi registrada de acordo com o questionamento direto ao paciente, em qualquer momento do estudo.^14^

O EEF foi considerado eficaz quando o estudo alcançou um dos objetivos: mínimo de 85% da FC máxima predita para a idade, calculada com a equação de Karvonen (FC máxima: 220 – Idade)^15^ ou presença de sinais ecocardiográficos de isquemia (novas alterações na mobilidade segmentar do VE).^11^

Os critérios de interrupção do teste considerados não diagnósticos foram: sintomas intoleráveis, hipertensão arterial reativa (pressão arterial sistólica >230 mmHg ou pressão arterial diastólica >120 mmHg), hipotensão relativa ou absoluta (queda da pressão sistólica >30 mmHg de repouso ou pressão arterial sistólica <80 mmHg), arritmias supraventriculares (taquicardia supraventricular sustentada e fibrilação atrial) e arritmias ventriculares (taquicardia ventricular não sustentada e sustentada).^16^

Os critérios de segurança do exame foram estabelecidos como aqueles que potencialmente causam risco de vida, definidos na metanálise publicada por Geleijnse et a. cols. como ruptura cardíaca, IAM, acidente vascular cerebral (AVC), assistolia, fibrilação ventricular (FV) e taquicardia ventricular sustentada (TVS).^17^ Foram definidos como efeitos colaterais menores angina, náuseas, cefaleia, hipertensão arterial reativa e hipotensão arterial (queda da PAS >30mmHg em relação a PAS de repouso e que necessita de reposição de cristaloide). Esses eventos não oferecem risco de vida, têm curta duração e são revertidos com a interrupção do exame, conforme definido pelo estudo de segurança de Wilson et al.^18^

Quanto às arritmias cardíacas registradas durante o exame, definiu-se: taquicardia paroxística supraventricular (TPSV), presença de complexos QRS estreitos (<120ms), na ausência de distúrbio de condução, regulares e semelhantes entre si; fibrilação atrial (FA), ausência de onda P associada com ritmo irregular, complexos QRS estreitos, na ausência de distúrbio de condução; extrassístoles ventriculares (EV), presença de complexos ventriculares prematuros e com frequência maior que 6 complexos por minuto; bigeminismo ventricular, presença de extrassístoles ventriculares alternadas com complexos QRS normais; taquicardia ventricular não sustentada (TVNS), presença de mais que três batimentos ventriculares complexos prematuros, com duração menor que 30 segundos e com frequência cardíaca maior que 100 batimentos por minuto (bpm); e taquicardia ventricular sustentada (TVS), presença de mais de três batimentos ventriculares complexos prematuros, com duração maior que 30 segundos e FC maior que 100bpm.^14^

O VE foi dividido em 17 segmentos miocárdicos, segundo as recomendações da ASE.^13 , 15^ A análise qualitativa da mobilidade segmentar miocárdica foi baseada na avaliação visual do espessamento miocárdico e no grau de mobilidade da parede graduada em um índice de mobilidade segmentar, dando a cada um dos segmentos a seguinte pontuação: 1 normal; 2 hipocinesia; 3 acinesia; e 4 discinesia. O valor normal desse índice é 1 (17 pontos/17 segmentos). Qualquer valor maior que 1 foi considerado índice de mobilidade segmentar alterado. Considerou-se exame positivo para isquemia miocárdica a presença nítida de alteração de mobilidade miocárdica segmentar em um ou mais segmentos do VE, durante o EEF.^11 , 13 , 14^

Nos pacientes do grupo 1, o ACE foi injetado em *bolus* , na dose de 0,5 a 1 mL em repouso, durante o protocolo e na fase de recuperação. A quantidade do ACE feita, durante o EEF, ficou a critério do médico ecocardiografista e teve como objetivo a completa opacificação das bordas endocárdicas durante o exame.^9^ Uma ampola de SonoVue^®^ foi utilizada em, no máximo, dois pacientes (proporção de 1:2), sempre respeitando as normas de esterilidade e no tempo entre exames, com intervalo inferior a 6h.^9^

As reções alérgicas ao SonoVue^®^ foram classificadas em:

- **Grau leve:** espirros, formigamento, urticária, prurido e dor costolombar, sem necessidade de tratamento medicamentoso
- **Grau moderado:** espirros, formigamento, urticária e prurido, com necessidade do uso de anti-histamínico e/ou corticosteroide
- **Grau acentuado:** sinais e sintomas de reação alérgica grave (choque anafilático), necessitando de tratamento imediato com epinefrina intramuscular, inalação de agonistas β-2 adrenérgicos para broncospasmos, anti-histamínico e corticosteroide.^12^

### Análise estatística

Os resultados foram apresentados em forma de tabelas e gráficos. As variáveis categóricas foram apresentadas em frequência e percentual. As variáveis contínuas foram apresentadas em mediana e intervalo interquartil. Na comparação entre os grupos para as variáveis categóricas, foram utilizados os testes Fisher e o qui-quadrado. Foi empregado o teste Kolmogorov-Smirnov para verificar a existência ou não de diferença significativa das variáveis contínuas que não apresentaram distribuição normal entre os grupos estudados. Este teste foi utilizado por se tratar de uma comparação entre os dois grupos, na qual as variáveis testadas não apresentaram distribuição normal, sendo, nesta situação, o teste mais sensível a qualquer diferença nas distribuições das quais se extraíram as amostras. Para todos os testes, foi usado nível de 95% do intervalo de confiança, sendo considerado o valor de p significativo menor que 0,05. Os dados foram analisados no programa estatístico Statistical Package for the Social Sciences 2.1 (SPSS).

Para o o cálculo amostral, foi utilizado como referência o estudo de segurança de Abdelmoneim e cols., em que foram avaliados 26.774 pacientes. Nesse estudo, ocorreram 94 óbitos em 30 dias; sendo assim, o cálculo amostral de proporção (amostras infinitas) foi estimado em 0,035109 (94/26.774), com erro de 0,25%.^20^ Em nosso estudo, o cálculo amostral foi de 2.150 pacientes.

## Resultados

Foram avaliados 2.346 pacientes, sendo 1.099 do grupo 1 e 1.247 do grupo 2. Perderam seguimento clínico 37 pacientes do grupo 1 (3%) e 73 do grupo 2 (5%). Portanto, a amostra final estudada foi de 1.062 (grupo 1) e 1.174 (grupo 2).

Os pacientes do grupo 1 eram predominantemente do gênero masculino e apresentavam índices de superfície corporal (ISC) e de massa corporal (IMC) maiores, conforme mostra a Tabela 1 .

**Tabela 1:** – Características gerais da amostra dos pacientes dos grupos 1 e 2

Variável	Com contraste (n=1.099) Mediana (Q1-Q3)	Sem contraste (n=1.247) Mediana (Q1-Q3)	p
Idade	65,0 (56,0-74,0)	65,0 (57,0-73,0)	0,460
Peso	84,0 (72,0-98,0)	74,0 (64,0-85,0)	<0,001*
Altura	167,0 (160,0-174,0)	164,0 (157,0-174,0)	<0,001*
ISC	1,90 (1,75-2,08)	1,80 (1,65-1,95)	<0,001*
IMC	30,0 (26,0-35,0)	27,4 (24,4-31,0)	<0,001*
Sexo masculino^1^	613 (55,8%)	511 (41,0%)	<0,001*

Testes de qui-quadrado e de Kolmogorov-Sminorv; * Significativo; Q1-Q3: intervalo interquartil das medianas; ISC: índice de superfície corporal; IMC: índice de massa corporal.

Um dado importante em nosso estudo é que o emprego do ACE permitiu a visibilização adequada de todos os segmentos do VE nos pacientes estudados, contribuindo para a melhora da qualidade do exame.

Observa-se também que o grupo 1 tinha maior número de pacientes hipertensos, obesos, sedentários e com maior frequência de angioplastia prévia. No grupo 2, havia mais pacientes ex-tabagistas e com história familiar para DAC. A Tabela 2 demonstra a distribuição da terapêutica antianginosa entre os grupos.

**Tabela 2:** – Distribuição dos pacientes quanto aos fatores de risco para DAC e terapêutica antianginosa nos grupos 1 e 2

Variáveis	Grupo 1 (n=1.099)	Grupo 2 (n=1.247)	p

Fatores de risco para DAC			
HAS	764 (69,52%)	812 (65,12%)	0,025*
DM	266 (24,20%)	276 (22,13%)	0,220
IAM prévio	93 (8,46%)	96 (7,70%)	0,495
Tabagismo	65 (5,91%)	73 (5,85%)	0,507
Ex-tabagismo	38 (3,46%)	342 (27,43%)	<0,001*
DLP	425 (38,67%)	524 (42,02%)	0,109
CVRM	30 (2,73%)	23 (1,84%)	0,164
ACTP	221 (20,11%)	153 (12,27%)	<0,001*
H. FamDAC	153 (13,92%)	309 (24,78%)	<0,001*
Obesidade	556 (50,59%)	391 (31,36%)	<0,001*
Sedentarismo	783 (71,25%)	790 (63,35%)	<0,001*
D. Chagas	8 (0,73%)	39 (3,13%)	<0,001*
**Terapêutica antianginosa**			
Betabloqueadores	151 (13,74%)	325 (26,06%)	<0,001*
Nitratos	0 (0,00%)	19 (1,52%)	<0,001*
Estatinas	214 (19,47%)	342 (27,43%)	<0,001*
Antiagregantes	198 (18,02%)	255 (20,45%)	0,136

Teste de Fisher; * Significativo HAS: hipertensão arterial sistêmica; DM: diabetes melito; IAM: infarto agudo do miocárdio; DLP: dislipidemias; CRVM: cirurgia de revascularização miocárdica; ACTP: angioplastia prévia; H. FamDAC: história familiar para doença arterial coronariana; D. Chagas: doença de Chagas.

Quanto aos parâmetros ecocardiográficos ( Tabela 3 ), foi observado que o grupo 1 apresentava valores de mediana discretamente maiores quando comparado ao grupo 2 para as seguintes variáveis: raiz da aorta (RaAo), átrio esquerdo (AE), volume do AE, dimensão diastólica final do ventrículo esquerdo (DDFVE), septo interventricular (SIV) e parede posterior do ventrículo esquerdo (PPVE).

**Tabela 3:** – Parâmetros ecocardiográficos hemodinâmicos, geométricos e funcionais dos grupos 1 e 2

Caracteristicas basais ecográficas e hemodinâmicas	Com contraste (n=1099)	Sem contraste (n=1.247)	p

	Mediana (Q1-Q3)	Mediana (Q1-Q3)	
RaAO	32,0 (29,0-35,0)	31,0 (28,0-34,0)	<0,001*
AE	37,0 (34,0-40,0)	35,0 (31,0-38,0)	<0,001*
Vol. AE	28,0 (23,0-30,0)	21,0 (18,0-27,0)	<0,001*
DDFVE	47,0 (44,0-51,0)	46,0 (43,0-50,0)	0,001*
DSFVE	29,0 (27,0-32,0)	29,0 (26,0-32,0)	0,053
FEVE	66,0 (61,0-70,0)	65,5 (60,0-70,0)	0,001*
SIV	9,0 (8,0-10,0)	8,0 (7,0-9,0)	<0,001*
PPVE	9,0 (8,0-10,0)	8,0 (7,0-9,0)	<0,001*
IEMSR	1,0 (1,0-1,0)	1,0 (1,0-1,0)	0,440
IEMSE	1,0 (1,0-1,0)	1,0 (1,0-1,0)	0,625
PAS	130,0 (120,0-140,0)	130,0 (120,0-130,0)	0,001*
PAD	80,0 (80,0-80,0)	80,0 (80,0-80,0)	<0,001*
FC	70,0 (63,0-78,0)	70,0 (64,0-70,0)	<0,001*

Teste de Kolmogorov-Sminorv; *Significativo; Q1-Q3: intervalo interquartil das medianas; AE: átrio esquerdo; Vol. AE: volume do átrio esquerdo; DDFVE: dimensão diastólica final do ventrículo esquerdo; DSFVE: dimensão sistólica final do ventrículo esquerdo; FEVE: fração de ejeção do ventrículo esquerdo; IEMSR: índice de escore de mobilidade segmentar em repouso; IEMSE: índice de escore de mobilidade segmentar no estresse; PAS: pressão arterial sistólica; PAD: pressão arterial diastólica; FC: frequência cardíaca.

Quanto à análise das arritmias apresentadas durante o exame, o grupo 2 apresentou maior incidência de EV isoladas e TPSV. Da mesma forma, nesse grupo, houve maior incidência de cefaleia e HA reativa durante o exame ( Tabela 4 ).

**Tabela 4:** – Incidência de arritmias, efeitos colaterais menores e eventos adversos induzidos durante o EEF nos grupos 1 e 2

Variáveis	Grupo 1 (n=1.099)	Grupo 2 (n=1.247)	p
**Arritmias induzidas durante o estresse farmacológico**			
EV	180 (16,4%)	247 (19,8%)	0,032*
ESV	74 (6,7%)	66 (5,3%)	0,162
TVNS	6 (0,5%)	10 (0,8%)	0,617
TVS	0 (0,0%)	1 (0,1%)	1,000
FV	0 (0,0%)	0 (0,0%)	-
TPSV	2 (0,2%)	15 (1,2%)	0,003*
FA	2 (0,2%)	2 (0,2%)	1,000
Bradicardia	1 (0,1%)	0 (0,0%)	0,469
**Efeitos colaterais menores**			
Angina	20 (1,8%)	14 (1,1%)	0,170
Cefaleia	5 (0,5%)	19 (1,5%)	0,012*
Náuseas	4 (0,4%)	8 (0,6%)	0,397
HA reativa	3 (0,3%)	19 (1,5%)	0,002*
Hipotensão arterial	1 (0,1%)	2 (0,2%)	1,000
Efeitos adversos	(n=1.062)	(n=1.174)	
Óbito com menos de 24h	0 (0,0%)	0 (0,0%)	-
Óbito com menos de 30 dias	0 (0,0%)	2 (0,17%)	0,276*
IAM com menos de 24h	0 (0,0%)	1 (0,1%)	0,525
IAM com menos de 30 dias	0 (0,0%)	0 (0,00%)	-

Teste de Fisher; *Significativo; EEF: ecocardiograma de estresse farmacológico; EV: extrassístole ventricular; ESV: extrassístole supraventricular; TVNS: taquicardia ventricular não sustentada; TVS: taquicardia ventricular sustentada; FV: fibrilação ventricular; TPSV: taquicardia paroxística supraventricular; FA: fibrilação atrial; HA: hipertensão arterial; IAM: infarto agudo do miocárdio.

**Figura 1:**
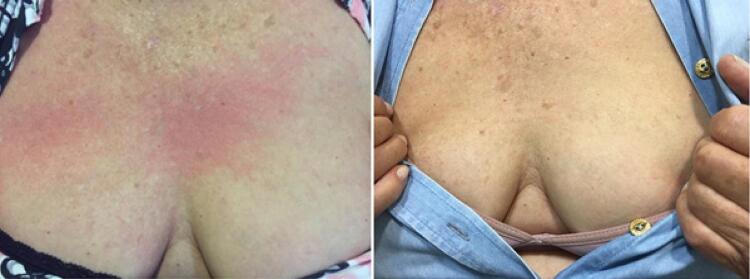
– *Exemplo de uma paciente com reação alérgica/urticária com uso de SonoVue^®^ e com melhora clínica após o uso do anti-histamínico oral.*

Foram observados eventos adversos como IAM e morte somente no grupo 2. Um paciente apresentou IAM com menos de 24h pós-exame. Houve dois óbitos com menos de 30 dias. O primeiro caso foi uma paciente de 80 anos de idade, com resultado positivo para isquemia miocárdica no EEF (multiarterial), evoluindo com IAM com menos de 24h pós-exame, necessitando de internação em unidade de terapia intensiva (UTI) e com falecimento no sétimo dia pós-exame. O segundo caso foi um óbito no 17º dia pós-EEF por causa não cardiovascular. As reações alérgicas encontradas no grupo 1 foram prurido e urticária. Todas elas em mulheres e de forma simultânea. A incidência total de reações alérgicas foi baixa (0,6%). Observou-se urticária em três (0,3%) dos pacientes, sendo dois casos de apresentação precoce (menos de 30min e com doses de 4,8mL) e um caso com apresentação tardia (após 24h com doses de 2,5mL do ACE), conforme demonstrado na Figura 1 e na Tabela 5 .

**Figura 2:**
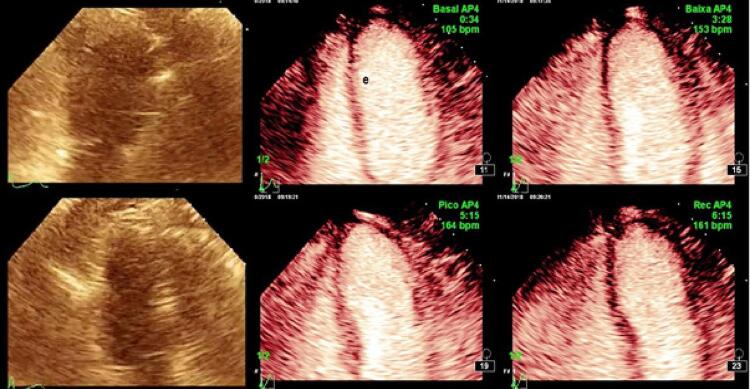
– *Paciente com janelas acústicas limitadas com melhora da imagem após o uso do agente de contraste ecocardiográfico.*

**Tabela 5:** – Distribuição das reações adversas ao ACE (SonoVue®) no grupo I durante o EEF

Grupo I (n=1.089) Variável	N	%
Reação alérgica	3	0,3
**30min após infusão**		
Prurido (n=1.086)	2	0,2
Espirros (n=1.083)	0	0,0
Urticárias (n=1.083)	2	0,2
Sibilos (n=1.083)	0	0,0
Reações anafiláticas (n=1.083)	0	0,0
Angioedema (n=1.085)	0	0,0
Choque anafilático (n=1.085)	0	0,0
**24h após infusão**		
Prurido (n=1.081)	1	0,1
Espirros (n=1.081)	1	0,1
Urticárias (n=1.082)	1	0,1
Sibilos (n=1.082)	0	0,0
Reações anafiláticas (n=1.082)	0	0,0
Angioedema (n=1.082)	0	0,0
Choque anafilático (n=1.082)	0	0,0

*N: Número de pacientes; h: horas; min: minutos; EEF: ecocardiograma sob estresse farmacológico.*

As doses de ACE administradas durante o EEF variaram de 1,5mL a 4,8mL. A dose de 1,5mL foi administrada em cinco pacientes (0,5%), 2,5mL em 913 (83,1%) e 4,8mL em 79 (7,2%).

Houve repetição do EEF com ACE com menos de 1 ano em 90 pacientes (8,5%). Dentre eles, uma paciente apresentou urticária com menos de 30min da infusão, com dose administrada de 4,8mL.

## Discussão

Esta coorte apresentou um número total de 2.346 pacientes, com predomínio do gênero masculino no grupo que recebeu ACE e com média de idade semelhante entre os grupos. Esses três dados estão de acordo com o estudo de segurança de Tsutsui et al.^19^ Nossa casuística foi menor quando comparada aos outros estudos de segurança com outros ACE já existentes.^1 , 20 - 28^ Dentre esses estudos, nossos dados foram semelhantes ao estudo de Abdelmoneim et al.,^20^ em que o grupo que recebeu ACE apresentou predomínio do gênero masculino e com IMC >30 kg/m^2^Os dois grupos apresentaram semelhança quanto aos FR para DAC, mas o grupo 2 tinha maior número de pacientes em uso contínuo de betabloqueadores (17,8 *vs* . 27,7 com p<0,001). Os pacientes do grupo 2 apresentaram maior incidência de cefaleia e HA reativa durante o EEF em comparação aos pacientes do grupo 1. O uso contínuo do betabloqueador, sem suspensão prévia, poderia justificar uma maior incidência desses efeitos colaterais citados durante o EEF com dobutamina, devido a um maior estímulo adrenérgico dos receptores alfa e bloqueio direto dos barorreceptores vasovagais, levando, consequentemente, a uma maior frequência de HA reativa e cefaleia.^19 , 22^

Em nosso estudo, houve maior incidência de EV e TPSV no grupo 2, no qual não foi empregado o ACE. Esse dado corrobora com a segurança do ACE na população estudada. O surgimento de arritmias durante o EEF está relacionado com a presença de disfunção ventricular, idade avançada, história prévia de arritmia e alterações da mobilidade segmentar em repouso.^17^ Esses fatores de risco são semelhantes nos dois grupos estudados, portanto, não podemos atribuir esses motivos como responsáveis por essa diferença em nosso estudo.^29^ Outra explicação poderia ser o emprego de uma maior dose de dobutamina durante o exame, visto que o grupo 2 apresentava maior número de pacientes em uso contínuo de betabloqueador.^30^ Contudo, não podemos confirmar essa hipótese, pois, infelizmente, não comparamos a dose de dobutamina empregada entre os grupos. Os dados do nosso estudo diferem dos encontrados por Saikh et al.,^23^ que demonstraram maior incidência de arritmias como EV, FA e TVNS no grupo que recebeu ACE.^23^ Em contrapartida, Abdelmoneim et al.^20^ observaram que houve semelhança na ocorrência de arritmias entre as coortes.^20^ Tsutsui et al.,^19^ não observaram diferença entre os seus dois grupos estudados quanto à incidência de TVNS, TVS ou TPSV.^19^

Quanto aos desfechos de IAM e morte, nossos dados assemelham-se ao estudo conduzido por Gabriel et al.,^22^ no qual os pacientes do grupo que recebeu ACE não apresentaram desfecho de mortes (0/0,0% *vs* . 2/0,04%).^22^

Shaikh et al.^23^ avaliaram, retrospectivamente, duas coortes e não observaram mortes entre os grupos.^23^ Vancraeynest et al.,^30^ descreveram em seu estudo um caso de IAM no grupo que recebeu ACE, sendo improvável a relação causal nesse caso. Esse estudo avaliou pacientes encaminhados para angiografia coronariana diagnóstica após realizarem ecocardiografia com ACE (albumina com dextrose aprimorada com perfluorcarboneto), com emprego de alto índice mecânico (1,5), utilizando o mesmo plano de imagem por 15min e com liberação subclínica de biomarcadores cardíacos. Foi observado que as imagens com baixos índices mecânicos (0,2) foram mais seguras.^30^

Na metanálise conduzida por Khawaja et al.^31^ envolvendo 211.162 pacientes, a mortalidade no grupo com ACE *versus* sem ACE foi 0,34% *versus* 0,9% com p=0,052, e a de IAM foi 0,15% *versus* 0,2% com p=0,72.^31^ Tais achados são semelhantes aos encontrados nos estudos de Dolan et al.,^1^ Abdelmoneim et al.,^20^ e Kunestzky et al.^26^ Nosso estudo apresentou incidência menor de IAM e morte quando comparado à metanálise citada anteriormente. Uma das razões para isso poderia ser o fato da nossa amostra ser constituída de pacientes ambulatoriais, estáveis, sem síndromes isquêmicas agudas ou qualquer situação crítica.

Alguns estudos, como de Tsutsui et al.,^19^ utilizando ACE como Optison^®^ e Definity^®^ e Aggeli et al.^28^ utilizando SonoVue^®^, não apresentaram eventos como IAM ou morte durante o EEF.

Diferentemente da nossa amostra ambulatorial, Anantharam et al.,^27^ demonstraram segurança dos ACE em pacientes submetidos à EEF com suspeita de síndrome coronariana aguda (SCA) estável. Durante um período de 4 anos, 3.704 pacientes foram submetidos à EEF ou ao ecocardiograma sob estresse com exercícios (EEE), sendo 929 (25%) pacientes com suspeita de SCA. Os agentes de contraste utilizados foram SonoVue^®^ (46%) e Luminity^®^ (54%) e não houve desfecho de morte, com ou sem ACE. Nesse mesmo estudo, não houve desfecho de IAM em pacientes que receberam ACE; em contrapartida, três pacientes no grupo sem contraste apresentaram IAM (p=0,24). Nosso estudo apresentou baixa incidência de reações alérgicas. Esse dado é semelhante ao encontrado por Aggeli et al.^28^ Nesse estudo, 23 (0,44%) pacientes, de um total de 5.250 que receberam SonoVue^®^, apresentaram prurido e urticária. Houve remissão do quadro com o uso de anti-histamínicos e sem necessidade de internação hospitalar.^28^

Wei et al.^21^ avaliaram, retrospectivamente, 78.383 pacientes e observaram que 0,01% da amostra apresentou eventos adversos graves, como provavelmente relacionados ao Definity^®^, dentro dos primeiros 30min após a administração, distribuídos igualmente entre homens e mulheres. Houve dois casos com reações alérgicas do tipo urticária e edema labial, mas sem anormalidades respiratórias, e que regrediram com o uso de anti-histamínico.^21^ Na metanálise de Khawaja et al.,^31^ nos 110.500 pacientes avaliados, a incidência de reações alérgicas e anafiláticas graves imediatamente após a administração do ACE foi de 0,009% e 0,004% respectivamente.^31^ Em outro estudo conduzido por Herzog et al.,^25^ a incidência de prurido e urticária foi de 2 (0,01%), e de reação anafilática 1 (0,01%).^25^ No estudo de Shaikh et al.,^23^ reação anafilática foi observada em um paciente (0,03%) após a administração do Definity^®^, sem exposição prévia ao contraste.^23^ Essas reações alérgicas muito raras e graves são secundárias a uma reação de hipersensibilidade do tipo 1, conhecida como pseudoalergia relacionada à ativação do complemento ou reações de CARPA.^12 , 32^

De acordo com Muskula et al.,^12^ a incidência de reações alérgicas com o emprego dos ACE ocorre em torno de 0,01%, e essas reações podem ser evitadas com a utilização de doses menores e com infusão lenta.^12^ Em nosso estudo, 83,1% dos pacientes receberam 2,5mL, e 7,2% receberam 4,8mL de SonoVue^®^. Dentro da nossa casuística, 8,5% dos pacientes refizeram o EEF com SonoVue^®^, em menos de 1 ano, e somente uma paciente apresentou urticária com menos de 30min, sendo, assim, difícil determinar a relação de dose-resposta.

### Limitações do estudo

1. Estudo unicêntrico, prospectivo, com pacientes ambulatoriais em que não foi incluído pacientes críticos ou em vigência de SCA.
2. Amostra com número de pacientes no limite inferior para análise de segurança do ACE.
3. Não houve comparação com outros agentes de contraste ecocardiográfico.

## Conclusão

SonoVue^®^ demonstrou segurança durante o EEF. Não ocorreram morte, IAM e reação anafilática durante o exame ou até 24h após a sua realização. Observou-se menor incidência de efeitos colaterais menores e arritmias no grupo que realizou EEF com ACE (SonoVue^®^) em relação ao grupo controle, assim como uma baixa incidência de reações alérgicas leves.
