# Supplementary material for: Segurança do Agente de Contraste SF6 (SonoVueⓇ) no Ecocardiograma sob Estresse Farmacológico
Source: Arq Bras Cardiol. 2021 Sep 16;117(6):1170–8. [Article in Portuguese] doi: 10.36660/abc.20200475 (PMC8757146; doi:10.36660/abc.20200475)
Supplement: Translation

# Translation: Safety of SF_6_(SonoVue^®^) Contrast Agent on Pharmacological Stress Echocardiogram

**Authors:** Rogerio Gomes Furtado, Daniela do Carmo Rassi, Luciano Henrique Melato, Ana Caroline Reinaldo de Oliveira, Paula Meneses Nunes, Priscila Elias Baccelli, Sara Camila de Oliveira Santos, Victor Emanuel Santos, Luiz Rassi, Colandy Godoy Nunes

**Affiliations:** 1 Brazil Centro de Diagnóstico por Imagem (CDI), Goiânia, GO – Brazil; 2 Brazil Universidade Federal de Goiás, Goiânia, GO – Brazil; 3 Brazil Hospital Santa Casa de Misericórdia de Goiânia, Goiânia, GO – Brazil

**Keywords:** Stress Echocardiogram, Echocardiographic Contrast Agent, Sonovue^®^, Safety

## Abstract

**Background:**

In 2007, the United States Food and Drug Administration mandated safety reviews of commercially available echocardiographic contrast agents (ECA), following reports of death. During the past years, different studies have proven the safety of ECA, but there have been few studies on SonoVue®.

**Objectives:**

To evaluate the safety of SonoVue® during pharmacological stress echocardiography (PSE), by analyzing the incidence of allergic reactions and comparing groups regarding the appearance of arrhythmia, minor side effects and adverse events.

**Methods:**

In this observational, prospective study, 2346 patients underwent PSE, and they were divided into the following 2 groups: group 1 with ECA (n = 1099) and group 2 without ECA (n = 1247). Patients were evaluated during PSE, at 24 hours, and at 30 days. Statistical significance was defined as p < 0.05.

**Results:**

Group 1 had fewer minor side effects, such as headache (5/0.5% versus 19/1.5%, p = 0.012) and less reactive hypertension (3/0.3% versus 19/1.5%, p = 0.002); fewer arrhythmias, such as ventricular extrasystoles (180/16.4% versus 247/19.8%, p = 0.032) and paroxysmal supraventricular tachycardia (2/0.2% versus 15/1.2%, p = 0.003); and no adverse events, such as acute myocardial infarction (AMI) or death. In group 2, 1 patient had AMI in < 24 hours (1/01%), and there were 2 deaths in < 30 days (2/0.1%). SonoVue®-related urticaria was seen in 3 (0.3%) patients, without anaphylactic reaction.

**Conclusion:**

SonoVue® demonstrated safety during PSE. No cases of death, AMI, or anaphylactic reaction were observed. There was a lower incidence of minor side effects and arrhythmias in the group that received ECA, as well as a low incidence of mild allergic reactions.

## Introduction

Echocardiography is recognized as a safe, non-invasive, and highly reproducible procedure for analyzing the anatomical and functional structures of the heart. However, up to 30% of exams face technical difficulties due to poor image quality,^1 , 2^ especially in patients who are obese, patients with thoracic deformities, and patients with chronic obstructive pulmonary disease.^3 , 4^

In 1997, the United States Food and Drug Administration (FDA) approved the use of echocardiographic contrast agents (ECA), with the aim of improving diagnostic accuracy of echocardiography, after reviewed data regarding the safety of ECA.^5^ Phase III trials demonstrated their safety, and, consequently, ECA were approved and released for endocardial border delineation.^6 , 7^

However, in October 2007, the FDA discontinued the use of ECA after 11 deaths that were temporally related to their use.^8^ Following review in 2008, the FDA once again approved the use of ECA, albeit with contraindications for patients with known intracardiac shunts or hypersensitivity to perflutren.^9^

The safety of ECA has been documented over the past years in diverse clinical scenarios, such as in patients with pulmonary hypertension, intracardiac shunts, and critical patients. Large studies have led to changes in FDA approval regarding the use of ECA in the described scenarios; moreover, the importance of their use in improving patient outcomes has been documented. Clinical trials have also demonstrated the safety and efficacy of ECA in physical and pharmacological stress echocardiography, as well as their use for evaluation of myocardial perfusion.^10^

Pharmacological stress echocardiography (PSE) is an established modality for diagnosis of coronary artery disease (CAD), whose safety has been demonstrated in several studies.^11^ The use of ECA on PSE has been consolidated over the years, initially, for endocardial border delineation and, subsequently, for evaluation of myocardial perfusion.^10^ The use of ECA is indicated when 2 or more segments of the left ventricle (LV) are not adequately visualized.^9 , 10^

In 2013, the Brazilian National Health Surveillance Agency (ANVISA, acronym in Portuguese) approved the use of SF_6_/sulfur hexafluoride (SonoVue®) in Brazil. While its safety has been previously demonstrated, there are few studies in the literature that report its use and the occurrence of adverse events.^12^

## Methods

### Study design

This observational, prospective, descriptive study was approved by the Research Ethics Committee of the Emergency Hospital of Goiânia (HUGO/protocol number 31442100 on Plataforma Brasil). Patients referred for risk stratification for CAD were evaluated by means of PSE. Patients were included after signing the informed consent form.

During PSE, when 2 or more LV segments were not adequately visualized, SonoVue® infusion was added for better delineation of the endocardial borders.^9 , 10^

Patients were divided into 2 groups. Group 1 comprised patients who underwent PSE with dobutamine-atropine and SonoVue® ECA, and group 2 comprised patients who underwent PSE with dobutamine-atropine, without any ECA.

Patients with history of allergic reaction to ECA were excluded from this study. Clinical and anthropometric data, risk factors for CAD, echocardiographic data, presence or absence of arrhythmias, adverse events, and allergic reactions within 30 minutes of the exam were obtained.

Patients in group 1 were clinically evaluated regarding signs and symptoms of allergic reaction during the first 30 minutes after the exam in person. After a 24-hour period, patients were evaluated in person or by telephone call.

In order to evaluate adverse events, such as acute myocardial infarction (AMI) and death, at 24 hours and 30 days, the researchers called all patients in both groups by telephone. Patients who did not answer the phone calls (3 calls on different days) and those who did not return to the cardiologists’ office or the diagnostic imaging center were excluded from the study.

### Echocardiography evaluation

PSE was carried out using EPIQ echocardiography devices (Philips Ultrasound Systems, Andover, MA, USA) . The exams were performed by echocardiographers who had received the same training, in a standardized and uniform manner, in accordance with the recommendations of the American Society of Echocardiography.^13^

Patients initially underwent baseline echocardiography, with acquisition of linear measurements of cardiac structures and valve flows. To evaluate left ventricular ejection fraction (LVEF), the Teichholz or Simpson methods were used, depending on the extent of change in segmental contraction. In some cases, end-systolic diameter was not measured when the Simpson method was used to calculate LVEF.^13 , 14^ Following acquisition of images in the baseline stage (parasternal longitudinal, transversal, apical 4-, 3- and 2-chamber planes), an intravenous infusion of dobutamine was initiated, with an initial dose of 5 µg/kg/min, with dose increments every 3 minutes at 10, 20, 30, and 40 µg/kg/min. Atropine was administered in doses of 0.25 mg, every minute, up to the maximum cumulative dose of 2 mg, in the event that patients did not show echocardiographic signs of myocardial ischemia and had not reached a heart rate of at least 100 bpm at the stage of 20 µg/kg/min.

For acquisition of specific images with ECA, the techniques of pulse-amplitude modulation and ultrasound pulse inversion (fundamental and harmonic) were used, with low mechanical index (< 0.20), associated or unassociated with a flash, to allow for uniform opacification of the endocardial boundary.^10^

A 30-minute monitoring period was standardized after the end of infusion, in order to evaluate the following: adverse effects, signs and symptoms of allergic reaction (group 1), and return of heart rate (HR) to a value below 100 beats per minute (bpm).^11^

During PSE, patients were kept under continuous monitoring (blood pressure, HR, and 12-lead electrocardiogram measurements). Symptoms were registered by directly questioning the patients, at any moment of the study.^14^

PSE was considered effective when the exam achieved 1 of the following objectives: at least 85% of the age-predicted maximal heart rate, calculated using Karvonen’s equation (maximal HR: 220 − age),^15^ or echocardiographic signs of ischemia (new alterations in LV segmental wall motion).^11^

The criteria for interrupting the exam, which were considered non-diagnostic, were the following: unbearable symptoms, reactive arterial hypertension (systolic blood pressure > 230 mmHg or diastolic blood pressure > 120 mmHg), relative or absolute hypotension (decrease of > 30 mmHg in relation to resting systolic pressure or systolic blood pressure < 80 mmHg), supraventricular arrhythmias (sustained supraventricular tachycardia or atrial fibrillation), and ventricular arrhythmias (non-sustained and sustained ventricular tachycardia).^16^

The safety criteria of the exam were established as the potentially life-threatening complications defined in the meta-analysis published by Geleijnse et al., such as cardiac rupture, AMI, stroke, asystole, ventricular fibrillation, and sustained ventricular tachycardia.^17^ Angina, nausea, headache, reactive arterial hypertension, and arterial hypotension (decrease of > 30 mmHg in relation to resting systolic blood pressure, requiring crystalloid replacement) were defined as minor side effects. These events are not life-threatening; they have a short duration, and they are reverted by interrupting the exam, as defined in the safety study by Wilson et al.^18^

Regarding cardiac arrhythmias registered during the exam, the following were defined: paroxysmal supraventricular tachycardia, presence of narrow QRS complexes (< 120 ms), in the absence of a conduction disorder, that were regular and similar to each other; atrial fibrillation, absence of P wave associated with irregular rhythm, narrow QRS complexes, in the absence of a conduction disorder; ventricular extrasystoles, presence of premature ventricular complexes, with a frequency higher than 6 complexes per minute; ventricular bigeminy, the presence of ventricular extrasystoles alternating with normal QRS complexes; non-sustained ventricular tachycardia, the presence of more than 3 premature ventricular contractions, lasting less than 30 seconds, with HR greater than 100 bpm; and sustained ventricular tachycardia, the presence of more than 3 premature ventricular contractions, lasting more than 30 seconds, and HR greater than 100 bpm.^14^

The LV was divided into 17 myocardial segments, in following with the recommendations of the American Society of Echocardiography.^13 , 15^ Qualitative analysis of segmental myocardial wall motion was based on visual evaluation of myocardial thickening and on the degree of wall motion graded on a segmental wall motion index, assigning the following scores to each segment: 1 normal; 2 hypokinesia; 3 akinesia; and 4 dyskinesia. The normal score on this index is 1 (17 points/17 segments). Any value greater than 1 was considered altered segmental wall score. A positive exam for myocardial ischemia was defined as the clear presence of altered segmental myocardial wall motion in 1 or more segments of the LV, during PSE.^11 , 13 , 14^

For patients in group 1, the ECA was injected as a bolus, at a dose of 0.5 to 1 ml at rest, during the protocol and the recovery phase. The amount of ECA applied during the PSE was at the discretion of the echocardiographer, with the aim of completely opacifying the endocardial borders during the exam.^9^ One ampoule of SonoVue® was used for a maximum number of 2 patients (1:2 ratio), consistently respecting sterility standards, with an interval of fewer than 6 hours between exams.^9^

Allergic reactions to SonoVue® were classified in the following manner:

- **Mild:** sneezing, tingling, urticaria, itching, and costolumbar pain, not requiring medical treatment;
- **Moderate:** sneezing, tingling, urticaria, and itching, requiring antihistamine and/or corticoid use;
- **Severe:** signs and symptoms of severe allergic reaction (anaphylactic shock), requiring immediate treatment with intramuscular epinephrine, inhalation of β-2 adrenergic agonists for bronchospasms, antihistamine, and corticoid drugs.^12^

### Statistical analysis

Results were shown as tables and graphs. Categorical variables were shown as frequency and percentage, and continuous variables were shown as median and interquartile range. For comparison of categorical variables between groups, Fisher’s test and the chi-square test were used. The Kolmogorov-Smirnov test was used to verify whether there was significant difference in continuous variables that did not show normal distribution between the study groups. This test was used because it was a comparison between both groups, where the tested variables did not show normal distribution; in this situation, it was the most sensitive test to any difference in distribution from which the samples were extracted. For all tests, a 95% confidence interval was applied, and p values less than 0.05 were considered significant. Data were analyzed using the statistics program Statistical Package for Social Sciences 2.1 (SPSS).

To calculate sample size, the safety study by Abdelmoneim et al., which evaluated 26,774 patients, was used as a reference. In that study, there were 94 deaths over 30 days, so the calculation of sample proportion (infinite samples) was estimated at 0.035109 (94/26,774), with an error of 0.25%.^20^ In our study, the sample size was calculated at 2150 patients.

## Results

This study evaluated 2346 patients, 1099 in group 1 and 1247 in group 2. Clinical follow-up was lost in 37 patients in group 1 (3%) and 73 in group 2 (5%). Thus, the final sample studied included 1062 patients in group 1 and 1174 in group 2.

Patients in group 1 were predominantly male, and they had higher body surface area and body mass indices, as shown in Table 1 .

**Table 1:** – General characteristics of the sample of patients in groups 1 and 2

Variable	With contrast (n = 1099) Median (Q1 – Q3)	Without contrast (n = 1247) Median (Q1 – Q3)	p
Age	65.0 (56.0 – 74.0)	65.0 (57.0 -73.0)	0.460
Weight	84.0 (72.0 – 98.0)	74.0 (64.0 – 85.0 )	< 0.001*
Height	167.0 (160.0 -174.0)	164.0 (157.0 – 174.0 )	< 0.001*
BSAI	1.90 (1.75 -2.08)	1.80 (1.65-1.95)	< 0.001*
BMI	30.0 (26.0 -35.0)	27.4 (24.4 -31.0)	< 0.001*
Male sex^1^	613 (55.8%)	511 (41.0%)	< 0.001*

Chi-square and Kolmogorov-Sminorv tests; * significant; Q1 – Q3: interquartile ranges of the median. BMI: body mass index; BSAI: body surface area index.

An important piece of data in our study is that the use of the ECA made adequate visualization of all LV segments possible in the studied patients, contributing to improved exam quality.

It was also observed that group 1 had a greater number of patients with hypertension, obesity, sedentarism, and higher frequency of prior angioplasty. In group 2, there were more patients who were former tobacco users and patients with family history of CAD. Table 2 shows the distribution of antianginal therapy between groups.

**Table 2:** – Distribution of patients by risk factors for coronary artery disease and antianginal therapy in groups 1 and 2

Variables	Group 1 (n = 1099)	Group 2 (n = 1247)	p

Risk factors for CAD			
SAH	764 (69.52%)	812 (65.12%)	0.025*
DM	266 (24.20%)	276 (22.13%)	0.220
Previous AMI	93 (8.46%)	96 (7.70%)	0.495
Tobacco use	65 (5.91%)	73 (5.85%)	0.507
Former tobacco use	38 (3.46%)	342 (27.43%)	< 0.001*
DLP	425 (38.67%)	524 (42.02%)	0.109
MRS	30 (2.73%)	23 (1.84%)	0.164
Prior angioplasty	221 (20.11%)	153 (12.27%)	< 0.001*
FHCAD	153 (13.92%)	309 (24.78%)	< 0.001*
Obesity	556 (50.59%)	391 (31.36%)	< 0.001*
Sedentarism	783 (71.25%)	790 (63.35%)	< 0.001*
Chagas disease	8 (0.73%)	39 (3.13%)	< 0.001*
**Antianginal therapy**			
Beta-blockers	151 (13.74%)	325 (26.06%)	< 0.001*
Nitrates	0 (0.00%)	19 (1.52%)	< 0.001*
Statins	214 (19.47%)	342 (27.43%)	< 0.001*
Antiplatelet agents	198 (18.02%)	255 (20.45%)	0.136

Fisher’s test; * significant. AMI: acute myocardial infarction; CAD: coronary artery disease; DM: diabetes mellitus; DLP: dyslipidemias; FHCAD: family history of coronary artery disease; MRS: myocardial revascularization surgery; SAH: systemic arterial hypertension.

With respect to echocardiography parameters ( Table 3 ), it was observed that group 1 had slightly higher median values, when compared to group 2, for the following variables: aortic root, left atrium, left atrial volume, left ventricular diastolic diameter, interventricular septum, and left ventricular posterior wall.

**Table 3:** – Hemodynamic, geometric, and functional echocardiographic parameters of groups 1 and 2

Echocardiographic and hemodynamic baseline characteristics	With contrast (n = 1099)	Without contrast (n = 1247)	p

	Median (Q1 – Q3)	Median (Q1 – Q3)	
AoR	32.0 (29.0 – 35.0)	31.0 (28.0 – 34.0)	< 0.001*
LA	37.0 (34.0 - 40.0)	35.0 (31.0 – 38.0)	< 0.001*
LAV	28.0 (23.0 – 30.0)	21.0 (18.0 – 27.0)	< 0.001*
LVDD	47.0 (44.0 – 51.0)	46.0 (43.0 – 50.0)	0.001*
LVSD	29.0 (27.0 – 32.0)	29.0 (26.0 – 32.0)	0.053
LVEF	66.0 (61.0 – 70.0)	65.5 (60.0 – 70.0)	0.001*
IVS	9.0 (8.0 – 10.0)	8.0 (7.0 – 9.0)	< 0.001*
LVPW	9.0 (8.0 – 10.0)	8.0 (7.0 – 9.0)	< 0.001*
RWMSI	1.0 (1.0 – 1.0)	1.0 (1.0 – 1.0)	0.440
SWMSI	1.0 (1.0 – 1.0)	1.0 (1.0 – 1.0)	0.625
SBP	130.0 (120.0 – 140.0)	130.0 (120.0 -130.0)	0.001*
DBP	80.0 (80.0 – 80.0)	80.0 (80.0 – 80.0)	< 0.001*
HR	70.0 (63.0 – 78.0)	70.0 (64.0 – 70.0)	< 0.001*

Kolmogorov-Sminorv test; * significant; Q1 – Q3: interquartile ranges of the median. AoR: aortic root; bpm: beats per minute; DBP: diastolic blood pressure; HR: heart rate; IVS: interventricular septum; LA: left atrium; LAV: left atrial volume; LVDD: left ventricular diastolic diameter; LVEF: left ventricular ejection fraction; LVPW: left ventricular posterior wall; LVSD: left ventricular systolic diameter; RWMSI: resting wall motion score index; SBP: systolic blood pressure; SWMSI: stress wall motion score index.

Regarding analysis of arrhythmias that presented during the exam, group 2 had a higher incidence of isolated ventricular extrasystoles and paroxysmal supraventricular tachycardia. Likewise, in group 2, there was a higher incidence of headache and reactive arterial hypertension during the exam ( Table 4 ).

**Table 4:** – Incidence of arrhythmias, minor side effects, and adverse events induced during PSE in groups 1 and 2

Variables	Group 1 (n = 1099)	Group 2 (n = 1247)	p
**Arrhythmias induced during pharmacological stress**			
VE	180 (16.4%)	247 (19.8%)	0.032*
SVE	74 (6.7%)	66 (5.3%)	0.162
NSVT	6 (0.5%)	10 (0.8%)	0.617
SVT	0 (0.0%)	1 (0.1%)	1.000
VF	0 (0.0%)	0 (0.0%)	-
PSVT	2 (0.2%)	15 (1.2%)	0.003*
AF	2 (0.2%)	2 (0.2%)	1.000
Bradycardia	1 (0.1%)	0 (0.0%)	0.469
**Minor side effects**			
Angina	20 (1.8%)	14 (1.1%)	0.170
Headache	5 (0.5%)	19 (1.5%)	0.012*
Nausea	4 (0.4%)	8 (0.6%)	0.397
Reactive AH	3 (0.3%)	19 (1.5%)	0.002*
Arterial hypotension	1 (0.1%)	2 (0.2%)	1.000
Adverse effects	(n=1062)	(n=1174)	
Death within 24 h	0 (0.0%)	0 (0.0%)	-
Death within 30 days	0 (0.0%)	2 (0.17%)	0.276*
AMI within 24 h	0 (0.0%)	1 (0.1%)	0.525
AMI within 30 days	0 (0.0%)	0 (0.00%)	-

Fisher’s test; * significant. AF: atrial fibrillation; AH: arterial hypertension; AMI: acute myocardial infarction; NSVT: non-sustained ventricular tachycardia; PSE: pharmacological stress echocardiogram; PSVT: paroxysmal supraventricular tachycardia; SVE: supraventricular extrasystole; SVT: sustained ventricular tachycardia; VE: ventricular extrasystole; VF: ventricular fibrillation.

Adverse events such as AMI and death were observed only in group 2. One patient had AMI fewer than 24 hours after the exam. There were 2 deaths in fewer than 30 days. The first case was an 80-year-old patient with a positive result for myocardial ischemia on PSE (multivessel). The patient progressed to AMI fewer than 24 hours after the exam, requiring hospitalization in an intensive care unit, and he died on the seventh day after the exam. The second case was a death on the seventeenth day after PSE, due to a non-cardiovascular cause. The allergic reactions found in group 1 comprised itching and urticaria. All of these cases occurred in women, in a simultaneous manner. The overall incidence of allergic reactions was low (0.6%). Urticaria was observed in 3 patients (0.3%), with 2 cases of early presentation (under 30 minutes, with 4.8-ml doses) and 1 case of late presentation (after 24 hours, with 2.5-ml doses of ECA), as shown in Figure 1 and Table 5 .

**Figure 1:** – *Example of a patient with allergic reaction/urticaria to use of SonoVue^®^, with clinical improvement after use of an oral antihistamine drug.*

**Figure 2:** – *Patient with limited acoustic windows, with improved imaged after use of the echocardiographic contrast agent.*

**Table 5:** – Distribution of adverse reactions to echocardiographic contrast agent (SonoVue®) in group I during PSE

Group I (n=1089) Variable	N	%
Allergic reaction	3	0.3
**30 min after infusion**		
Itching (n=1086)	2	0.2
Sneezing (n=1083)	0	0.0
Urticaria (n=1083)	2	0.2
Wheezing (n=1083)	0	0.0
Anaphylactic reactions (n=1083)	0	0.0
Angioedema (n=1085)	0	0.0
Anaphylactic shock (n=1085)	0	0.0
**24 h after infusion**		
Itching (n=1081)	1	0.1
Sneezing (n=1081)	1	0.1
Urticaria (n=1082)	1	0.1
Wheezing (n=1082)	0	0.0
Anaphylactic reactions (n=1082)	0	0.0
Angioedema (n=1082)	0	0.0
Anaphylactic shock (n=1082)	0	0.0

*N: Número de pacientes; h: horas; min: minutos; EEF: ecocardiograma sob estresse farmacológico.*

Doses of ECA administered during PSE ranged from 1.5 ml to 4.8 ml. The dose of 1.5 ml was administered in 5 patients (0.5%); 2.5 ml in 913 (83.1%); and 4.8 ml in 79 (7.2%).

PSE with ECA was repeated within less than 1 year in 90 patients (8.5%). Of these patients, 1 had urticaria less than 30 minutes after infusion, with an administered dose of 4.8 ml.

## Discussion

This cohort included a total number of 2346 patients. Patients were predominantly male in the group that received ECA, and mean age was similar between the groups. These 3 pieces of data are in agreement with the safety study by Tsutsui et al.^19^ Our sample size was smaller than that of other safety studies on other existing ECA.^1 , 20 - 28^ Among these studies, our data were similar to those of the study by Abdelmoneim et al.,^20^ where the group that received ECA was predominantly male, with body mass index > 30 kg/m^2^ .^20^ Both groups were similar in terms of risk factors for CAD, but group 2 had a greater number of patients on continuous use of beta-blockers (17.8 versus 27.7 with p < 0.001). Patients in group 2 showed a higher incidence of headache and reactive arterial hypertension during PSE, when compared to patients in group 1. Continuous use of beta-blockers, without prior suspension, could justify a higher incidence of these side effects mentioned during PSE with dobutamine, due to higher adrenergic stimulation of alpha receptors and direct block of vasovagal baroreceptors, consequently leading to a higher frequency of reactive arterial hypertension and headache.^19 , 22^

In our study, there was a greater incidence of paroxysmal supraventricular tachycardia in group 2, where ECA was not used. This piece of data corroborates the safety of ECA in the study population. The appearance of arrhythmias during PSE is related to the presence of ventricular dysfunction, advanced age, previous history of arrhythmia, and alterations in resting segmental wall motion.^17^ These risk factors were similar in both study groups; therefore, it is not possible to consider these motives as responsible for this difference in our study.^29^ Another explanation could be that a higher dose of dobutamine was used during the exam, given that, in group 2, there was a greater number of patients on continuous use of beta-blockers.^30^ We cannot, however, confirm this hypothesis, because, unfortunately, we did not compare the dobutamine doses used between the groups. Data from our study differ from those found in the study by Saikh et al.,^23^ which demonstrated a higher incidence of arrhythmias, such as ventricular extrasystole, atrial fibrillation, and non-sustained ventricular tachycardia in the group that received the ECA.^23^ In contrast, Abdelmoneim et al.^20^ observed that there was a similarity in the occurrence of arrhythmias between their cohorts.^20^ Tsutsui et al.^19^ found no difference between their 2 study groups regarding the incidence of non-sustained ventricular tachycardia, sustained ventricular tachycardia, or paroxysmal supraventricular tachycardia.^19^

Regarding the outcomes of AMI and death, our data are similar to those of the study conducted by Gabriel et al.,^22^ where the outcome of death did not occur in patients in the group that received the ECA (0/0.0% versus 2/0.04%).^22^

Shaikh et al.^23^ retrospectively evaluated 2 cohorts, and they did not observe any deaths between the groups.^23^ Vancraeynest et al.^30^ described, in their study, a case of AMI in the group that received ECA, but a causal relationship was unlikely in this case. Their study evaluated patients referred for diagnostic coronary angiography after undergoing echocardiography with ECA (perfluorocarbon-enhanced dextrose albumin), using a high mechanical index (1.5), with the same imaging plane for 15 minutes and subclinical release of cardiac biomarkers. It was observed that images with low mechanical indices (0.2) were safer.^30^

In the meta-analysis conducted by Khawaja et al.,^31^ involving 211,162 patients, the mortality in the group that received ECA versus the group without ECA was 0.34% versus 0.9%, with p = 0.052, and that of AMI was 0.15% versus 0.2%, with p = 0.72.^31^ These findings are similar to those found in the studies by Dolan et al.,^1^ Abdelmoneim et al.,^20^ and Kunestzky et al.^26^ Our study showed a lower incidence of AMI and death when compared to the aforementioned meta-analysis. One of the reasons for this could be the fact that our sample consisted of outpatients who were stable, without acute ischemic syndromes or critical situations.

Some studies, for instance, Tsutsui et al.,^19^ using Optison® and Definity® as ECA, and Aggeli et al.,^28^ using SonoVue®, did not find any events, such as AMI or death, during PSE.

Differently from our sample of outpatients, Anantharam et al.^27^ demonstrated the safety of ECA in patients undergoing PSE with suspected stable acute coronary syndrome. Over a 4-year period, 3,704 patients underwent PSE or exercise stress echocardiography; 929 (25%) of these patients had suspected acute coronary syndrome. The ECA used were SonoVue® (46%) and Luminity® (54%), and no deaths occurred in the groups with or without ECA. In this same study, there were no outcomes of AMI in patients who received ECA; on the other hand, 3 patients in the group without ECA had AMI (p = 0.24).^27^ Our study showed a low incidence of allergic reactions. These data are similar to those found by Aggeli et al.^28^ In their study, 23 (0.44%) patients out of a total of 5250 who received SonoVue® showed itching and urticaria. The condition was reverted with the use of antihistamines, without requiring hospitalization.^28^

Wei et al. retrospectively evaluated 78,383 patients, and they observed that 0.01% of the sample had severe adverse events, considered probably related to Definity®, within the first 30 minutes after administration, distributed equally between men and women. There were 2 cases of allergic reaction such as urticaria and lip edema, but there were no respiratory abnormalities and all patients recovered after use of an antihistamine drug.^21^

In the meta-analysis by Khawaja et al.,^31^ which evaluated 110,500 patients, the incidence of severe allergic and anaphylactic reactions immediately after administration of ECA was 0.009% and 0.004%, respectively.^31^ In another study conducted by Herzog et al.,^25^ the incidence of itching and urticaria was 2 (0.01%), and that of anaphylactic reaction was 1 (0.01%).^25^ In the study by Shaikh et al.,^23^ anaphylactic reaction was observed in 1 patient (0.03%) after administration of Definity®, without prior exposure to contrast.^23^ These very rare and severe allergic reactions are secondary to a type 1 hypersensitivity reaction known as complement activation-related pseudo-allergy or CARPA.^12 , 32^

According to Muskula et al.,^12^ the incidence of allergic reactions with the use of ECA occurs in approximately 0.01% of cases, and these reactions can be avoided by using lower doses with slow infusion.^12^ In our study, 83.1% of patients received 2.5 ml, and 7.2% received 4.8 ml of SonoVue®. In our sample, 8.5% of patients repeated PSE with SonoVue®, in under 1 year, and only 1 patient showed urticaria in under 30 minutes, thus making it difficult to determine the dose-response relationship.

### Study limitations

1. This was a prospective, single-center study with outpatients, and it did not include critical patients or patients with acute coronary syndrome
2. The number of patients in the sample was at the lower limit for safety analysis of ECA.
3. Comparisons were not made with other ECA.

## Conclusions

SonoVue® demonstrated safety during PSE. No cases of death, AMI, or anaphylactic reaction occurred during the exam or within 24 hours after it was performed. A lower incidence of minor side effects and arrhythmias was observed in the group that underwent PSE with SonoVue® ECA, in comparison with the control group, and there was a low incidence of mild allergic reactions.
